# *Momordica charantia*-Derived Extracellular Vesicles Provide Antioxidant Protection in Ulcerative Colitis

**DOI:** 10.3390/molecules28176182

**Published:** 2023-08-22

**Authors:** Feng Wang, Meng Yuan, Chenqi Shao, Nan Ji, Haifeng Zhang, Chunmei Li

**Affiliations:** 1 College of Tourism and Culinary Science, Yangzhou University, Yangzhou 225009, China; 13142695810@163.com (F.W.); yuanmengzy1234@163.com (M.Y.); 18115091111@163.com (C.S.); swordart364518@gmail.com (N.J.); 2 Key Laboratory of Chinese Cuisine Intangible Cultural Heritage Technology Inheritance, Ministry of Culture and Tourism, Yangzhou University, Yangzhou 225009, China

**Keywords:** extracellular vesicles, *Momordica charantia*, protein domain analysis, antioxidant, ulcerative colitis

## Abstract

Plant-derived extracellular vesicles are functional nanovesicles that have significant applications in both disease prevention and treatment, as well as for use as drug carriers. *Momordica charantia* is a widely consumed food that has both medicinal and nutritional properties and has shown intervention in diabetes and inflammation caused by oxidative damage. In this study, *Momordica charantia*-derived extracellular vesicles (MCEVs) were extracted and demonstrated to have excellent antioxidant activity by characterization, lipid composition analysis, protein domain analysis, and in vitro antioxidant measurement. In addition, in vivo studies indicated that the MCEVs could restore ulcerative colitis by regulating oxidation and inflammatory factors. Therefore, the antioxidant properties of MCEVs may be important in protecting the colon from inflammation, which provides new insights into the application of MCEVs as drugs or vectors for intervention in ulcerative colitis.

## 1. Introduction

Ulcerative colitis (UC) is a chronic, nonspecific intestinal disease characterized by diarrhea, abdominal pain, weight loss, and bloody stools with intestinal inflammation and destruction of the intestinal epithelium [1,2]. It is common in young adults aged 30–40 years and can cause severe disability [3]. Recently, the incidence of colitis has been increasing globally, with a projected prevalence of 0.1% by 2030, double that in 2010 [4]. Studies have indicated that the pathogenesis of colitis may be related to an inflammatory reaction, oxidative damage, intestinal barrier dysfunction, or imbalance of intestinal microbiota, but the exact etiology and mechanism are still unclear [5,6]. Maintaining and relieving chronic inflammation are the main objectives of current treatment [7]. Both surgical and conventional medical treatment have side effects, such as causing potential systemic toxicity, gastrointestinal adverse reactions, and postoperative complications [8,9]. Studies have found that oral administration preferentially focuses on the site of inflammation and is more conducive to site-targeted therapy than intravenous administration [10]. Therefore, scientists are keen to develop oral drugs that target the inflammatory areas of the gut without side effects.

Plant-derived extracellular vesicles (PEVs) are widely used in pharmaceuticals for their unique functions. Studies have shown that they can serve as carriers for targeted drug delivery, maximize drug efficacy, and prolong pharmacological activity [11]. Interspecies communication can be achieved by the induction of a variety of cytokines [12]. Moreover, PEVs have innate therapeutic potential, with functional activity such as antioxidant, anti-inflammatory, anti-tumor, etc., which may change the treatment of various diseases in the future [13,14,15]. Gao et al. showed that turmeric nanovesicles had anti-inflammatory activity that could restore the damaged intestinal epithelial barrier by reducing the expression of inflammatory factors and the abundance of Bacteroides, Escherichia-Shigella, Helicobacter, and Staphylococcus and then alleviate the symptoms of chronic colitis induced by DSS [16]. Exosome-like nanoparticles from mulberry bark have been shown to activate the AhR signaling pathway, increase COPS8 expression, and reduce cullin-1 deneddylation activity. These could be used as potential drugs for the prevention and treatment of intestinal diseases. [17]. Similarly, extracellular vesicles from grapefruit and tomato could more effectively transport the foreign protein Hsp70 to glioma cells; upregulate the expression of HO-1, NAO1, GCLM, and GCLC; inhibit the production of IL-2; and regulate the antioxidant response of human cells [18]. Therefore, plant-derived food nanoparticles with unique properties have potential application prospects in the targeted therapy of colitis.

*Momordica charantia* (MC), commonly known as bitter melon or bitter gourd, mainly contains carbohydrates (49 g kg^−1^ edible portion), crude protein (20.9 g kg^−1^), dietary fiber (14 g kg^−1^), vitamin C (780 mg kg^−1^), etc. [19,20]. It has been shown to have anti-oxidative, anti-inflammatory, anti-cancer, and hypoglycemic effects and has been described as a panacea for inflammation and cancer [21,22]. At present, researchers have studied the regulatory effects of MC homogenates, polysaccharides, and seed extracts on UC, but there are no reports on MC-derived extracellular vesicles (MCEVs). Studies by Semiz et al. showed that MC-homogenized jam could reduce the inflammation in rat colon tissue [23]. And MC polysaccharides improved intestinal permeability and inhibited intestinal inflammation by inactivating NF-κB signaling [24]. Proteins from MC seeds exhibited antioxidant effects, which can reduce inflammatory factors in vitro by regulating oxidative stress but aggravate inflammatory symptoms in vivo [25]. This may be related to the route of intraperitoneal administration. Thus, we hypothesized that the outer vesicles of balsam pear might also have a UC effect. In this study, we will extract the vesicles of MC by differential centrifugation. First, proteomics and lipid composition identification will be performed to identify the potential antioxidant and anti-inflammatory potential of MCEVs. Then, 1,1-diphenyl-2-picryl-hydrazyl (DPPH) and hydroxyl scavenging effects will be measured to verify the antioxidant activity in vitro. Finally, the DSS-induced moue UC model will be verified by gavage to prove that MCEVs can regulate UC through antioxidation. It is expected that this study will provide a theoretical basis for treating ulcerative colitis and provide a new direction for developing *Momordica charantia*.

## 2. Results and Discussion

### 2.1. MCEV Characterization

**Figure 1:**
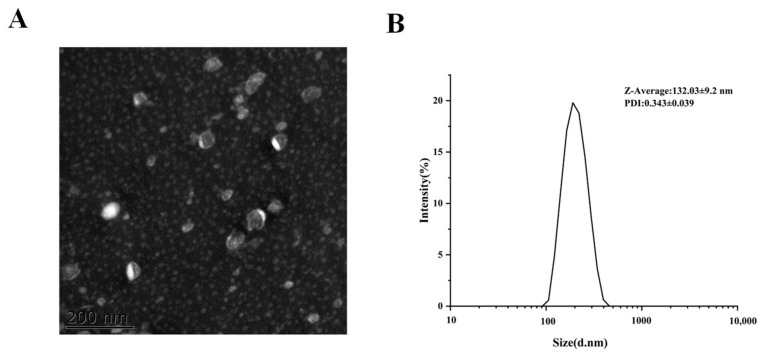
Characterization of MCEVs. (**A**) Transmission electron microscopic (TEM) images of MCEVs. (**B**) The size of MCEVs.

Particle size, polydispersity index (PDI), and potential are very important characteristics of nanoparticles that can affect toxicity, stability, and biological distribution [26]. Similar to the exterior in Cui’s sport, the MCEVs in this study presented a cup shape, as shown in Figure 1, with an average particle size of 132.03 nm in the reported range of 106–147 nm [27,28]. PDI showed excellent uniformity at 0.34. In addition, the potential was −11.19 ± 3.07 mV, which was nearly neutral. All the results indicated that the MCEVs were effective for further study.

### 2.2. Lipid Composition Analysis of MCEVs

The structure of lipids is diverse, consisting of three levels with a broad class, subclass, and molecule. The function of lipids depends on the subclass of lipids, and to a certain extent, the type and content of the subclass can reflect the changes in lipid function [29]. According to Table 1, a total of 19 lipid subclasses were identified in the MCEVs. The analysis of the relative proportion of lipids showed that sphingolipids accounted for the highest amount of total lipids at 66.34%, of which Sphingosine SPH constituted 55.01%. Phospholipids accounted for 17.95%, of which PE (8.23%) and PC (6.36%) were relatively high. Glycerol was mainly composed of TG (7.46%). This was consistent with the results of Subra’s study. The lipid composition of exosomes was similar to that of cell membranes and was mainly composed of cholesterol, phospholipids, and sphingolipids [30]. Compared with ordinary cell membranes, exosome membranes were more mobile, indicating that the MCEVs had potential as carriers and transport mediums [31]. Additionally, PE, PG, and Ps were predominantly present on the mitochondrial membrane, and these acetal phospholipids all play an active role in antioxidation, effectively triggering the phagocytosis and clearance behavior of cells [32].

### 2.3. Protein Domain Analysis of MCEVs

**Figure 2:**
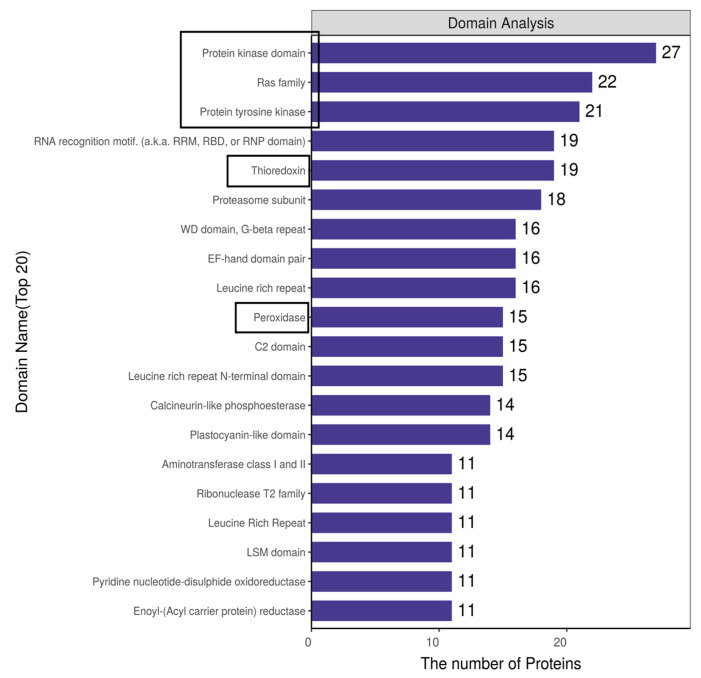
Domain analysis of MCEVs.

Protein domains are key functional units for protein biological functions. The top 20 domains involved in MCEV protein kinase are shown in Figure 2. Among these domains, the protein kinase domain plays a major regulatory role in almost every aspect of cell biology [33]. Protein tyrosine kinase activates protein kinase, and the kinase domain also catalyzes the phosphorylation of other tyrosine residues, creating docking sites for adaptor proteins or enzymes that lead to downstream signaling. It plays an important role in targeted cancer therapy. For example, epidermal growth factor receptor (EGFR) is a protein kinase, and overexpression of EGFR can lead to cancer. Colitis and breast cancer can be treated by targeting the protein kinase domain to regulate AKT or Ras signaling downstream of the protein tyrosine kinase receptor. [34]. Ras can enhance RAS-GTP signaling and promote cell proliferation and transformation, which plays an important role in tumor therapy [35]. Thioredoxin is an antioxidant involved in the pathway regulation of various protein interactions to manipulate the dynamic regulation of the structure and function of downstream proteins. It can directly reduce lipid hydrogen peroxide, catalyze the denitrification of cytoplasmic caspase-3, suppress STAT3 and JNK signaling, and ultimately induce cancer cell apoptosis. [36]. In addition, peroxidase is associated with inflammation and oxidative stress [37]. Therefore, some of the proteins of MCEVs have antioxidant capacity, which may relieve the symptoms of the disease through its antioxidant effect in disease treatment.

### 2.4. GO Annotation and KEGG Analysis of MCEVs

**Figure 3:**
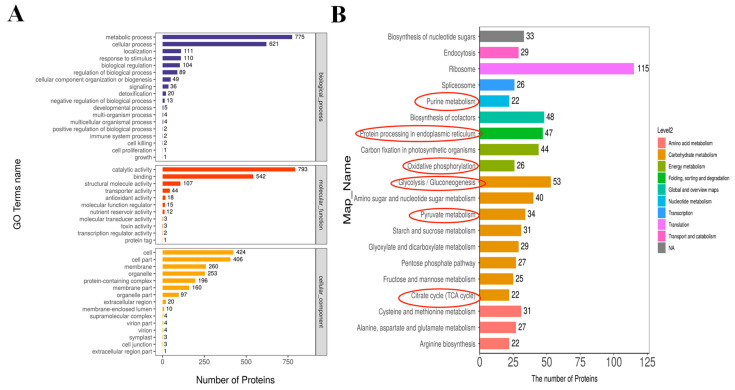
GO annotate and KEGG pathway annotation. (**A**) GO annotate statistics (level 2). (**B**) KEGG pathway annotation statistics (top 20).

The top 20 GO functional annotations of MCEVs proteins were mainly divided into biological processes, molecular functions, and cellular components, involving metabolic processes, cellular processes, localization, stimulus–response, biological regulation, and regulation of biological processes (Figure 3A). They primarily played functions of catalytic activity, binding, structural molecular activity, transport activity, antioxidant activity, and molecular function regulation and were chiefly involved in the composition of cells, cell parts, membranes, and organelles. Both molecular function and protein domain annotations implied the provincial antioxidant function of MCEVs.

The top 20 KEGG analyses of MCEV proteins were involved in pathways of the ribosome, glycolysis/gluconeogenesis, biosynthesis of cofactors, carbon fixation in photosynthetic organisms, amino sugar and nucleotide sugar metabolism, and protein processing in the endoplasmic reticulum (Figure 3B). Therefore, glycolysis and gluconeogenesis, pyruvate metabolism, purine metabolism, and TCA cycle pathways are related to metabolic disorders and impact obesity, diabetes, and other diseases [38]. The endoplasmic reticulum pathway is considered to be a checkpoint for protein folding, causing cellular stress associated with inflammation, cancer, and diabetes diseases when an abnormal accumulation of unfolded or misfolded proteins occurs [39,40]. Oxidative phosphorylation is associated with necrotizing enterocolitis, which alleviates inflammation by regulating autophagy [41]. Therefore, MCEVs can be considered potential natural replenishment agents to improve obesity, diabetes, inflammation, and cancer.

### 2.5. Antioxidant Activity of MCEVs In Vitro

**Figure 4:**
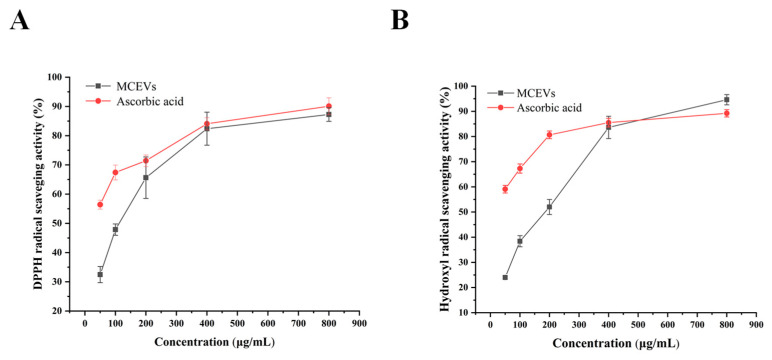
Antioxidant activity of MCEVs. (**A**) The DPPH free radical scavenging activity of MCEVs. (**B**) The hydroxyl radical scavenging activity of WRNPs. Ascorbic acid (Vc) was used as a positive control.

The determination of DPPH free radical scavenging activity has been widely used to evaluate the antioxidant effect of natural compounds. The prominent DPPH free radical scavenging activity signifies the great ability of the substance to provide electrons or hydrogen [42]. Hydroxyl radical is a kind of reactive oxygen species (ROS) that is in homeostasis in normal physiological metabolism. Once the intrinsic defense system is damaged or excess free radicals are present, oxidative stress will occur [43]. The previous lipid composition and proteomics of MCEVs showed potential antioxidant activity, such as acetal phospholipids [32], thioredoxin [36] and peroxidase [37]. Thus, the DPPH method and hydroxyl radical scavenging methods were used to prove the antioxidant activity of MCEVs. The DPPH free radical and hydroxyl free radical scavenging rates of MCEVs were dose dependent (Figure 4). When the protein concentration of MCEVs reached 400 μg/mL, the DPPH free radical and hydroxyl radical scavenging activities were not significantly different from those of the positive control ascorbic acid. When the protein concentration of MCEVs reached 800 μg/mL, the hydroxyl radical scavenging activity of 94.64 ± 2.04% was significantly higher than that of Vc (89.23 ± 1.46). Aljohi’s study [44] showed that the DPPH free radical scavenging activity of 3.2 mg/mL *Momordica charantia* polysaccharide was 56%. Chen’s study [45] showed that the DPPH free radical scavenging activity of 15 mg/mL *Momordica charantia* pulp was 40% and the hydroxyl removal rate was 68%. Therefore, compared with *Momordica charantia* polysaccharide and *Momordica charantia* pulp, MCEVs have a greater ability to regulate oxidative stress.

### 2.6. MCEVs Ease UC in C57BL/6 Mice

**Figure 5:**
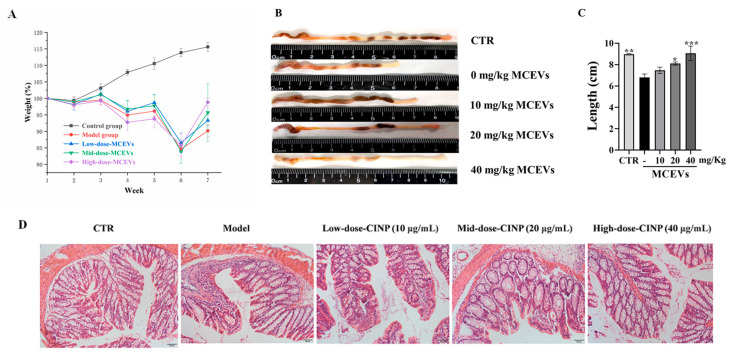
The chronic colitis model-building method and the effect of AST@PLGA in mice. (**A**) The changes of body weight among different groups. (**B**,**C**) The changes in mouse colon length and shape. (**D**) The histopathological changes of mouse colon tissue; data are presented as mean ± standard deviation. *p*-values < 0.05 were regarded as statistically significant (* *p* < 0.05, ** *p* < 0.01, *** *p* < 0.001) compared with the 0 mg/Kg MCEV group.

UC is characterized by recurrent occurrence, so we constructed a UC model with multiple cycles to investigate the influence of MCEVs on enteritis. As shown in Figure 5, after three cycles induced by DSS, the weight of the mice declined drastically (Figure 5A), and the mice appeared to move slowly, be listless, and have matte hair, diarrhea, and bloody stool. After interfering with different concentrations of MCEVs, the body weight of mice increased, and the level of increase increased with increasing doses (Figure 5A). The anatomical analysis of the intestinal tract of mice (Figure 5B,C) showed that the length of the colon gradually recovered with the increase in MCEV dose, and the length of MCEVs of 40 mg/kg was significantly longer than that of the control (*p* < 0.05). This shows that a high dose of MCEVs can effectively alleviate intestinal damage caused by colitis. Pathological analysis (Figure 5D) showed that goblet cells were absent and neutrophils were aggregated in the model group. However, after interfering with different concentrations of MCEVs, anatomical analysis reduced symptoms of goblet cell loss and inflammatory cell infiltration. The above results indicate that the model of UC was successfully constructed in this study and that MCEVs can alleviate the symptoms of UC, so further experiments could be carried out.

### 2.7. MCEVs Inhibit Oxidative Stress Induced by UC

**Figure 6:**
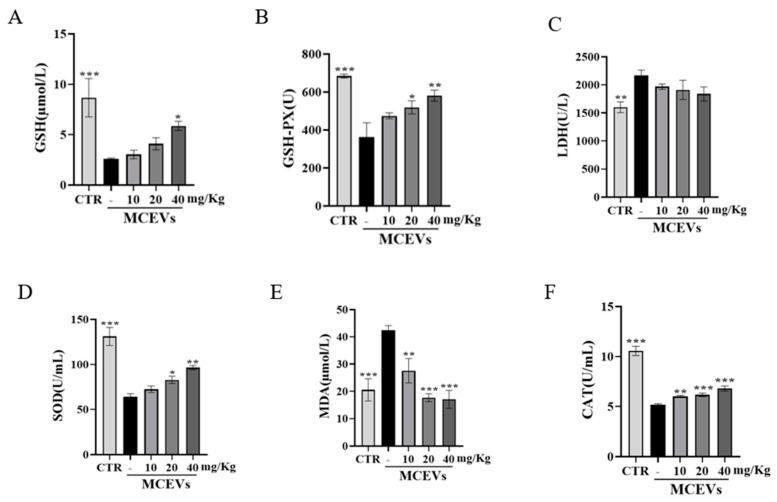
Changes in serum indexes in mice. (**A**) The content of GSH. (**B**) The content of GSH-PX. (**C**) The content of LDH. (**D**) The content of SOD. (**E**) The content of MDA. (**F**) The content of CAT; data are presented as mean ± standard deviation. *p*-values < 0.05 were regarded as statistically significant (* *p* < 0.05, ** *p* < 0.01, *** *p* < 0.001) compared with the 0 mg/Kg MCEV group.

Oxidative stress and inflammation have been documented as being closely related [46]. Jiang et al. indicated that when colitis occurs, the colon produces many oxygen-free radicals, which destroy the intestinal mucosal barrier [47]. Some enzymes in the body are involved in oxidative regulation. Superoxide dismutase (SOD) promotes the removal of free radicals and reduces lipid peroxidation. At the same time, glutathione peroxidase (GSH-PX) can remove H_2_O_2_ and effectively reduce •OH to decrease the damage. Catalase (CAT) is an indispensable enzyme in the biological antioxidant enzyme system because through its catalysis, excess H_2_O_2_ in the body can be decomposed into H_2_O and O_2_, thereby protecting cells and tissues from oxidative damage [48]. To assess the effect of MCEVs on oxidative stress, the levels of glutathione (GSH), SOD, catalase (CAT), GSH-Px, lactate dehydrogenase (LDH), and malondialdehyde (MDA) were measured in serum. Compared with the model group, MCEVs upregulated the levels of GSH, GSH-PX, SOD, and CAT and downregulated the levels of LDH and MDA (Figure 6), which is consistent with the findings of Chen et al. [49]. These results are also supported by the proteomics data mentioned above. It is possible that thioredoxin and peroxidases in MCEVs upregulate SOD, GSH, GSH-Px, and CAT levels, thereby inhibiting lipid oxidation and scavenging hydrogen peroxide. The results also exhibited that MCEVs could alleviate the oxidative damage caused by UC.

### 2.8. MCEVs Stimulate Inflammatory Factors in UC

**Figure 7:**
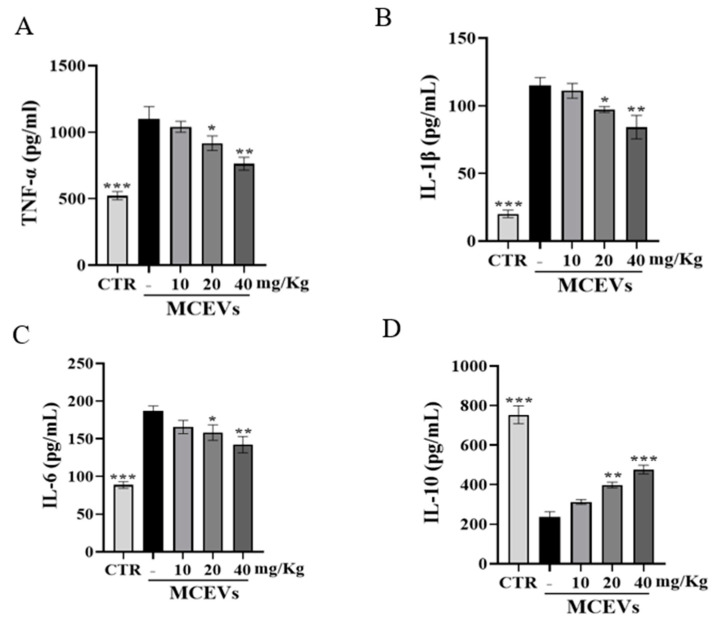
Changes in serum indexes in mice. (**A**) The content of proinflammatory cytokines TNF-α. (**B**) The content of proinflammatory cytokines IL-1β. (**C**) The content of proinflammatory cytokines IL-6. (**D**) The content of proinflammatory cytokines IL-10; data are presented as mean ± standard deviation. *p*-values < 0.05 were regarded as statistically significant (* *p* < 0.05, ** *p* < 0.01, *** *p* < 0.001) compared with the 0 mg/Kg MCEV group.

Excessive reactive oxygen species are normally produced in chronic intestinal inflammation, causing cell damage, which in turn aggravates inflammatory cell infiltration and inflammatory injury [50]. We have shown that MCEVs can reduce the level of oxidative stress in colitis. To further evaluate the effect of MCEVs on the intestinal inflammatory response, proinflammatory cytokines in serum were measured in this study. TNF-α, IL-1β, and IL-6 are key intestinal proinflammatory cytokines that play an important role in the pathogenesis and progression of UC [51]. When intestinal inflammation occurs, TNF-α is overproduced, resulting in intestinal damage. Studies have shown that excessive production of reactive oxygen species can activate NF-κB and further upregulate the expression of TNF-α and IL-1β [52]. IL-10 is an anti-inflammatory factor that can inhibit inflammation, and when colitis occurs, the expression of IL-10 is reduced [53]. MCEVs significantly inhibited the levels of IL-1β, IL-6, and TNF-α and advanced the level of IL-10 in mouse serum (Figure 7). This phenomenon was consistent with the Mytilus couscous polysaccharide-treated colitis mice, indicating that MCEVs can alleviate the symptoms of colitis and intestinal injury [54]. These results suggested that regulation of inflammatory factors might be an important property to improve UC through antioxidant protection of MCEVs, but the specific mechanism needs to be further studied.

## 3. Materials and Methods

### 3.1. Materials

*Momordica charantia* was purchased from the local market; phosphate buffer solution (PBS), DPPH and hematoxylin–eosin were purchased from Beyotime (Shanghai, China); methanol solution, 30% H_2_O_2_ solution, FeSO_4_, salicylic acid, paraffin, and 4% paraformaldehyde solution were purchased from SINOPHARM (Beijing, China); dextran sodium sulfate (DSS) was purchased from MP Biomedicals (Santa Ana, CA, USA); the detection kit for CAT, SOD, LDH, and MDA was purchased from Beyotime (Shanghai, China); the detection kits for GSH and GSH-PX were purchased from Jiancheng (Nanjing, China); and the ELISA kits for IL-6, IL-10, IL-1β, and TNF-α were purchased from BOSTER (San Mateo, CA, USA).

### 3.2. Preparation of MCEVs

*Momordica charantia* L. was washed with ultra-pure water and squeezed. The filtered juice was separated and extracted by differential centrifugation at 500× *g* for 10 min, 2000× *g* for 20 min, 5000× *g* for 30 min, and 10,000× *g* for 1 h at 4 °C. Finally, the supernatant was obtained and centrifuged at an ultrahigh speed of 150,000× *g* at 4 °C for 2.5 h, and 1 mL phosphate buffer solution was added to the precipitate, suspended again, and stored at −80 °C for use.

### 3.3. MCEV Characterization

One mL of MCEVs diluted 50 times was put into a potential dish and a particle-size dish, and the potential, PDI, and particle size were measured in a Marvin particle size analyzer [26].

Ten μL MCEVs were placed in copper mesh for 15 min, then stained with 10 μL 2% phosphotungstic acid for 1 min. After drying under an infrared lamp, the nanoparticles were observed under a Tecnai 12 transmission electron microscope.

### 3.4. Lipid Composition Identification

An appropriate amount of MCEVs was added to the standard mixture of 200 μL distilled water and 20 μL lactone, then 800 μL methyl tert-butyl ether and 240 μL pre-cooled methanol were successively added to the mixture. An ultrasound was performed on the obtained mixture in a water bath at low temperature for 20 min and centrifugated at room temperature for 30 min. After centrifugation, the upper organic phase was dried by a removal sample concentrator. The sample was separated by the UHPLC Nexera LC-30A (Shimadzu, Japan) ultra-high-performance liquid chromatography system and detected by electrospray ionization (ESI) in positive and negative ion modes. Finally, LipidSearch software was used for peak identification, peak extraction, and lipid identification (secondary identification) of lipid molecules and internal standard lipid molecules.

### 3.5. Proteomic Identification

The protein in MCEVs was extracted by lysis buffer, and the concentration was determined by a BCA kit. After digestion by pancreatic enzymes, the extracted peptide was desalted, concentrated by vacuum centrifuge, and redissolved in 40 μL 0.1% formic acid solution, and then 1 μL of the resolution was used for LC-MS/MS analysis.

LC-MS/MS analysis was performed on a Q Exactive (Thermo Scientific, Shanghai, China) mass spectrometer. Then, the MS raw data for each sample were combined and searched using the MaxQuant 1.5.3.17 software for identification and quantitation analysis. After that, protein sequences were searched using the InterProScan software 5.0 to identify protein domain signatures from the InterPro member database Pfam. The protein sequences of the selected differentially expressed proteins were locally searched using the NCBI BLAST+ client software (ncbi-blast-2.2.28+-win32.exe) and InterProScan to find homologous sequences. Then, gene ontology (GO) terms were mapped and sequences were annotated using the software program Blast2GO. The GO annotation results were plotted by R scripts. Following the annotation steps, the studied proteins were blasted against the online Kyoto Encyclopedia of Genes and Genomes (KEGG) database (http://geneontology.org/, accessed on 10 December 2022) to retrieve their KEGG orthology identifications and were subsequently mapped to pathways in KEGG.

### 3.6. Determination of DPPH Radical Scavenging Activity

The determination of the DPPH radical scavenging activity of MCEVs was described by Chen’s method with some modifications [55]. MCEVs were prepared in liquid with protein concentrations of 50, 100, 200, 400, and 800 mg/mL. Then, 100 µL of sample solution was added to 50 µL DPPH ethanol solution (0.1 mmol/mL) and incubated at 37 °C for 30 min, and absorbance A_1_ was measured at 515 nm. Under the same conditions, the absorbance A_2_ was measured with 50 µL ethanol instead of DPPH, and the absorbance A_3_ was measured with 100 µL phosphate buffer solution instead of MCEVs. Ascorbic acid was used as the positive control. The DPPH radical scavenging rate was calculated as follows:

DPPH free radical scavenging rate (%) = [1 − (A_1_ − A_2_)/A_3_] × 100%

### 3.7. Determination of Hydroxyl Radical Scavenging Activity

Hydroxyl radical scavenging activity was measured according to the Chen’s report [56]. First, absorbance A_1_ was measured at 510 nm after 50 µL MCEVs, 50 μL FeSO_4_ aqueous solution (2.25 mmol/L), 50 µL salicylic acid methanol solution (9 mmol/L), and 50 μL of H_2_O_2_ methanol solution (8.80 mmol/L) were mixed and kept at 37 °C for 30 min. Absorbance A_2_ was measured with 50 µL methanol instead of MCEVs. Absorbance A_0_ was measured with 100 µL methanol instead of H_2_O_2_ methanol and salicylic acid methanol solution. Ascorbic acid was used as the positive control. Hydroxyl free radical scavenging was calculated as follows:

Hydroxyl radical scavenging rate (%) = [1 − (A_1_ − A_2_)/A_0_] × 100%

### 3.8. Animals and Treatments

Specific-pathogen-free (SPF) male C57BL/6 mice, 6 weeks old, were acquired from the Comparative Medicine Center of Yangzhou University (Yangzhou, China) and raised in the standard mouse room. Animal studies were approved by the Jiangsu Administrative Committee for Laboratory Animals (license number: SYXK(SU) 2022-0044).

The mice were divided into five groups (n = 5/group). The mice in the control group were fed pure water to eat freely. Mice in treated groups were fed 2% DSS in the 1st, 3rd, and 5th weeks and pure water for the 2nd, 4th, and 6th weeks. Six weeks later, pure water, DSS (10, 20, 40 mg/kg), and MCEVs in a dose of 100 µL were given to control, modern (low, medium, high), and MCEV groups, respectively. After continuous gavage for 5 days, all the mice were anesthetized and killed, and the length of the colon was measured.

### 3.9. Histopathologic Analysis of Colon Tissue

An intestinal segment of about 5 mm was taken from the middle colon and dehydrated in 4% paraformaldehyde solution for 24 h, paraffin embedded, and then sliced into 4–5 μm sections. Hematoxylin–eosin (HE) staining was performed for observation, and the sections were observed after photographing under a microscope [57].

### 3.10. Determination of Antioxidant Indexes and Inflammatory Cytokines in Serum

The supernatant of mouse plasma was obtained after centrifugation at 3000× *g* for 10 min. The contents of CAT, SOD, LDH, MDA, GSH, and GSH-PX in serum were determined according to the requirements of the kit (Beyotime Biotechnology and Jiancheng, Shanghai, China). The levels of IL-6, IL-10, IL-1β, and TNF-α in serum were detected by ELISA kits (BOSTER, San Mateo, CA, USA).

### 3.11. Statistics

Each experiment was performed for at least three repetitions. The results were presented as mean ± standard deviation. Statistical mean differences were evaluated using SPSS 26.0. The results were considered statistically significant if *p* < 0.05.

## 4. Conclusions

In summary, MCEVs were prepared successfully and their characterization was determined. The lipid composition and proteomics analysis of MCEVs have demonstrated their capacity for antioxidant activity, suggesting their potential as a natural therapeutic agent for addressing conditions such as obesity, diabetes, inflammation, and cancer. Our in vitro antioxidant experiment involving MCEVs revealed no significant disparity in the scavenging rates of DPPH free radicals and hydroxyl free radicals compared to Vc at a protein concentration of 400 μg/mL. This finding provides additional evidence supporting the notable antioxidant activity of MCEVs and its potential as a remedy for diseases induced by oxidative stress. Further experiments of mice with UC showed that MCEVs could protect the colonic mucosa by regulating the oxidation and inflammation indexes in the blood of mice and alleviate the symptoms of colonic ulceration. Compared with the model group, MCEVs upregulated the levels of GSH, GSH-PX, SOD, and CAT but downregulated the levels of LDH and MDA. At the same time, MCEVs significantly inhibited the levels of IL-1β, IL-6, and TNF-α and advanced the level of IL-10 in mouse serum. This suggests that antioxidant protection is an important means for MCEVs to act on UC and that MCEVs may be a potential drug or carrier that could be applied to treat various oxidative stress-induced diseases.

## Figures and Tables

**Table 1:** Lipid subclass identification of MCEVs.

Lipid Subclass Names	Lipid Subclass Abbreviations	Content (%)
Sphingosine	SPH	55.01
Ceramide	Cer	11.01
Phosphatidylethanolamines	PE	8.23
Triglyceride	TG	7.46
Phosphatidylcholine	PC	6.36
Diglyceride	DG	5.21
Phosphatidylglycerols	PG	1.79
Zymosteryl	ZyE	1.41
Wax ester	WE	0.84
Monoglyceride	MG	0.72
Phosphatidylinositols	PI	0.6
Phosphatidylserines	PS	0.42
Sphingomyelin	SM	0.32
Cardiolipin	CL	0.29
Phosphatidic acid	PA	0.071
Lyso-phosphatidylcholine	LPC	0.07
Lyso-phosphatidylethanolamine	LPE	0.055
Lyso-phosphatidylinositol	LPI	0.039
Coenzyme	CO	0.011

## Data Availability

The datasets used and/or analyzed during the current study are available from the corresponding author on reasonable request.

## Sample Availability

Not available.
